# Book Reviews

**Published:** 1903-10

**Authors:** 


### *BOOK REVIEWS.*

---

Consumption a Curable and Preventable Disease. What a layman should know about it. By Laurena F. Flyck, M.D., Philadelphia. David McKay, Publisher, 1022 Market Street, Philadelphia. Price $1.

The object of this book is to acquaint the layman in language he can understand of the scientific facts connected with the subject of consumption. There has been much unnecessary fear on the score of communicability of the disease on the part of the public, and much misconception of its nature and course. The author strives to present in a popular manner the gist of our present knowledge of tuberculosis, in order to aid the crusade against it. The numerous questions which arise in connection with the disease are considered fully in the book. It seems destined to be of great service in promulgating a correct knowledge among the laity of the great white plague.

---

Progressive Medicine. A quarterly digest of advances, discoveries and improvements in the medical and surgical sciences. Edited by Hobart A. Hare, M.D., assisted by H. R. M. Landis, M.D. Vol. 3, September, 1903. Lea Bros. & Co., Philadelphia and New York.

This volume of Progressive Medicine is devoted to diseases of the thorax and its viscera, including the heart, lungs and blood-vessels, dermatology and syphilis, diseases of the nervous system, obstetrics. The first group of subjeets is treated by William Ewart, of London. Dermatology and Syphilis, well illustrated articles by W. S. Gottheil. Diseases of the Nervous System, by W. G. Spiller, Obstetrics, by R. C. Vorns. The volume is a handsome one, like its predecessors, and the subject new and fresh and up to date.

